# Fully Magnetically Polarized Ultrathin La_0.8_Sr_0.2_MnO_3_ Films

**DOI:** 10.1021/acsami.3c14031

**Published:** 2024-01-12

**Authors:** Federico Stramaglia, Gyanendra Panchal, Frithjof Nolting, Carlos A. F. Vaz

**Affiliations:** Swiss Light Source, Paul Scherrer Institut, Villigen 5232, Switzerland

**Keywords:** mixed-valence manganites, ultrathin films, oxide heterostructures, antiferromagnetic spintronics, molecular beam epitaxy, complex oxides

## Abstract

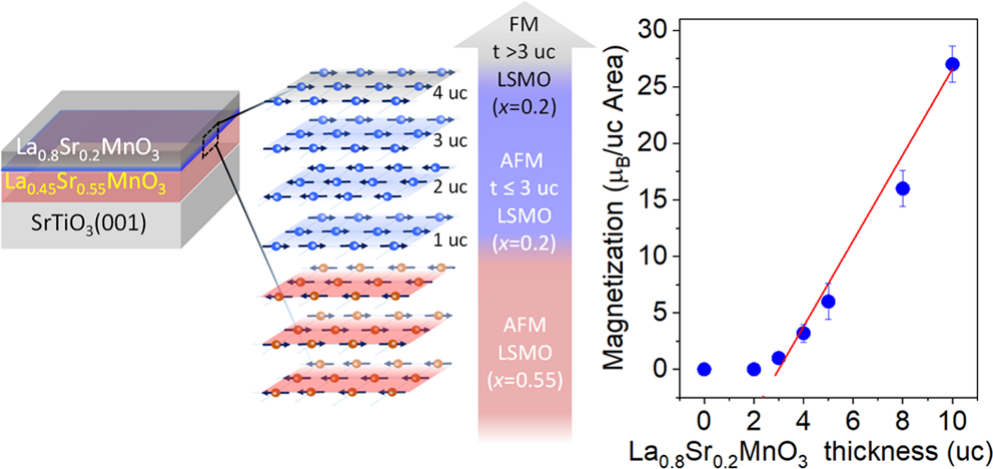

We report the observation of fully magnetically polarized ultrathin La_0.8_Sr_0.2_MnO_3_ films by using LaMnO_3_ and La_0.45_Sr_0.55_MnO_3_ buffer layers grown epitaxially on SrTiO_3_(001) substrates by molecular beam epitaxy. Specifically, we show that La_0.8_Sr_0.2_MnO_3_ films grown on 12-unit-cell LaMnO_3_ have bulk-like magnetic moments starting from a single unit cell thickness, while for the 15-unit-cell La_0.45_Sr_0.55_MnO_3_ buffer layer, the La_0.8_Sr_0.2_MnO_3_ transitions from an antiferromagnetic state to a fully spin-polarized ferromagnetic state at 4 unit cells. The magnetic results are confirmed by X-ray magnetic circular dichroism, while linear dichroic measurements carried out for the La_0.8_Sr_0.2_MnO_3_/La_0.45_Sr_0.55_MnO_3_ series show the presence of an orbital reorganization at the transition from the antiferromagnetic to ferromagnetic state corresponding to a change from a preferred in-plane orbital hole occupancy, characteristic of the A-type antiferromagnetic state of La_0.45_Sr_0.55_MnO_3_, to preferentially out of plane. We interpret our findings in terms of the different electronic charge transfers between the adjacent layers, confined to the unit cell in the case of insulating LaMnO_3_ and extended to a few unit cells in the case of conducting La_0.45_Sr_0.55_MnO_3_. Our work demonstrates an approach to growing ultrathin mixed-valence manganite films that are fully magnetically polarized from the single unit cell, paving the way to fully exploring the unique electronic properties of this class of strongly correlated oxide materials.

## 1 Introduction

The disruption of the atomic structure at the boundary between different materials often gives rise to the emergence of new phenomena that are characteristic of the interface region itself.^1,2^ The study of such interface phenomena is important to understanding the role of broken symmetries, electron exchange, and correlation effects to the electronic properties, but may also hold promise for new electronic devices based on purely interfacial effects. Critical for strong interfacial effects is the growth of sharp and well-defined interfaces separating materials with pristine electronic structures; this is because the presence of defects at the interface may strongly disturb the electronic properties of the component phases, for instance, making the interdiffused interface of a magnetic material nonmagnetic^3^ or where the presence of charge traps screens the polarization of a ferroelectric interface.^4^

One example of an interfacial effect is the onset of a magnetoelectric coupling emerging at the boundary between ferromagnetic and ferroelectric materials to form multiferroic heterostructures, whereby control of magnetism through electric fields is made possible.^4−10^ For the particular case of the Pb(Zr_0.2_Ti_0.8_)O_3_/La_0.8_Sr_0.2_MnO_3_ interface, the magnetoelectric coupling was demonstrated to originate from charge-driven modulation of the valency and spin configuration of the interfacial La_0.8_Sr_0.2_MnO_3_ layer induced by charge screening of the ferroelectric polarization.^11^ The large magnetoelectric coupling engineered in this system exploited the rich functional properties and complex phase diagrams of the mixed-valence manganites characterized by multiple electronic ground states.^12−16^ The observed changes in the magnetic moment of the La_0.8_Sr_0.2_MnO_3_ layer are on the order of 20%, which is a large effect; however, given the interfacial character of the magnetoelectric coupling, the change in the magnetic moment is associated with only a few unit cells of the whole sample, and therefore, the total change is averaged out by the remainder of the magnetic film. The latter has a lower thickness limit determined by the magnetic and electric behaviors of La_1–*x*_Sr_*x*_MnO_3_ thin films: when grown on high-quality SrTiO_3_(001) substrates, the onset of electric conductivity occurs at about 10 unit cells (uc), while the magnetic moment is strongly reduced and is absent below 5 uc,^17,18^ forming magnetic and electric “dead layers”, whose origin has been variously attributed to magnetic or orbital interfacial reconfiguration, cation intermixing, or polar discontinuity.^19−29^ It is therefore important to devise solutions for obtaining ultrathin films with bulk-like or enhanced interfacial characteristics, including full magnetic and ferroelectric polarizations in ferromagnetic and ferroelectric systems, respectively. In addition, one also expects that the relative importance of the interfacial effect will increase as the thickness is reduced since the interface will then be a larger portion of the overall system. Hence, it is important that not only the interface is well-defined but also that the electronic properties of the materials remain robust with decreasing thickness, ideally down to the monolayer range.^3,4^

To overcome the degradation in the magnetic properties of ultrathin manganites, one strategy consists of decoupling the films from the nonmagnetic substrate by inserting a magnetically polarized buffer layer.^20,30^ For example, the introduction of 2 uc LaMnO_3_ between a 120 nm thick La_1–*x*_Sr_*x*_MnO_3_ (*x* = 0.4) and the SrTiO_3_ substrate leads to the disappearance of the dead layer as measured by second harmonic signal generation and ascribed to the interruption of charge transfer across the SrTiO_3_ interface, which is otherwise responsible for hole doping of the La_0.6_Sr_0.4_MnO_3_ film.^20^ In another instance, 1 uc La_1–*x*_Ba_*x*_MnO_3_(*x* = 0.3) films sandwiched between two 3 uc SrRuO_3_ layers are found to be fully magnetically polarized, an effect attributed to the presence of oxygen octahedral rotations induced by SrRuO_3_, leading to an orbital reconstruction and an enhancement of the interfacial magnetic properties.^31^ In another example, CaRu_1/2_Ti_1/2_O_3_ used as an interlayer in La_2/3_Ca_1/3_MnO_3_ superlattices has been shown to preserve the magnetic properties of the individual manganite films and to result in antiferromagnetic interlayer coupling.^32^ These studies highlight the importance of separating the manganite film from the supporting SrTiO_3_ substrate and highlight the role of charge transfer, spin exchange, and octahedral tilting, together with orbital rearrangement, in the improvement of the magnetic properties toward bulk characteristics.

In this study, we explore the magnetic and transport properties of ultrathin La_0.8_Sr_0.2_MnO_3_ films when a manganite buffer layer is introduced at the interface with the SrTiO_3_ substrate. We employ two different types of buffer layers, a nominally antiferromagnetic insulating LaMnO_3_ and an antiferromagnetic conducting La_0.45_Sr_0.55_MnO_3_, in order to disentangle the roles of spin and charge exchange at the interface. In the first case, our results show that the La_0.8_Sr_0.2_MnO_3_ film develops robust magnetic properties, with a near-bulk-like critical temperature and magnetic moments that are independent of thickness starting at 1 uc. In the second case, an antiferromagnetic coupling to the buffer layer is observed for thicknesses up to 3 uc, while for larger thicknesses, the top La_0.8_Sr_0.2_MnO_3_ layer transitions to a fully polarized ferromagnetic state. By probing the electronic band structure using X-ray absorption spectroscopy, we link the magnetic properties of La_0.8_Sr_0.2_MnO_3_ to the evolution of orbital occupancy with film thickness.

## 2 Experimental Section

**Figure 1:**
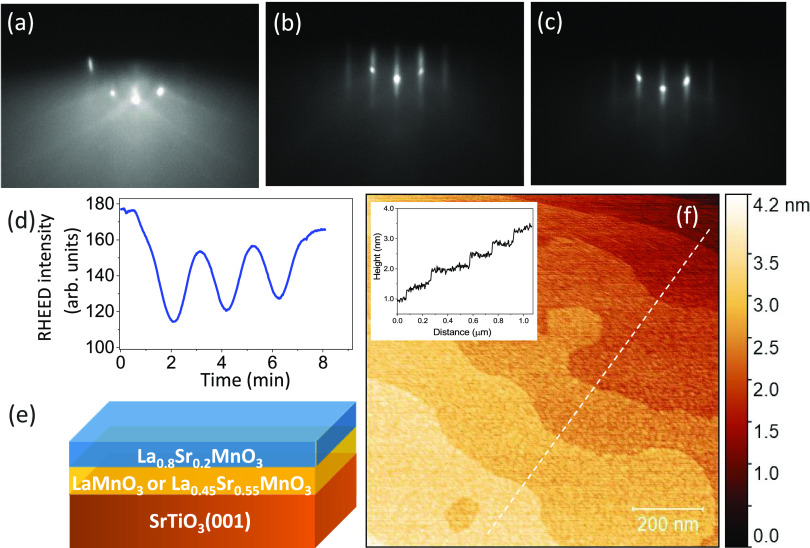
(a–c) RHEED patterns of the TiO_2_-terminated SrTiO_3_(001) substrate, the LaMnO_3_ buffer layer, and the 3 uc La_0.8_Sr_0.2_MnO_3_/LaMnO_3_ film, respectively, along the ⟨100⟩ azimuth. The energy of the incident electron beam was set to 15 keV. (d) RHEED intensity oscillations as a function of time for 3 uc La_0.8_Sr_0.2_MnO_3_ deposited on LaMnO_3_. (e) Schematic of the sample structure. (f) AFM measurement of the 10 uc of La_0.8_Sr_0.2_MnO_3_/LaMnO_3_ sample, showing a flat surface with single unit cell step terraces (inset).

The samples in this study consist of La_0.8_Sr_0.2_MnO_3_ films with thicknesses ranging from 0 to 10 uc, grown on a 12 uc LaMnO_3_ buffer layer (except for the 3 uc La_0.8_Sr_0.2_MnO_3_ sample, grown on 14 uc LaMnO_3_) or on a 15 uc La_0.45_Sr_0.55_MnO_3_ buffer layer. Figure 1e shows a schematic diagram of the sample structure. The buffer layer thickness is chosen based on previous works showing that 12 uc LaMnO_3_ is insulating and has a reduced magnetic moment^33^ and that 15 uc La_0.45_Sr_0.55_MnO_3_ is conducting antiferromagnetic.^34^ The samples were grown in an oxide molecular beam epitaxy (MBE) system with a base pressure of 2 × 10^–10^ mbar, equipped with five effusion cells, a reflection high-energy electron diffraction (RHEED) system, and a load-lock chamber for sample transfer. For this study, we used commercially available SrTiO_3_(001) substrates (SurfaceNet), with ±0.1° miscut, which were chemically treated with a HCl:HNO_3_ solution and annealed to 1000 °C in air to provide single TiO_2_-terminated surfaces.^35,36^ For metal oxide deposition, we set the molecular oxygen partial pressure to (5 ± 1) × 10^–7^ mbar, the substrate temperature to 720 °C, and the evaporation rate to about 0.5 uc/min, values that we found to provide the optimal growth conditions. In all cases, we adopted the same procedure for preparing the SrTiO_3_ substrate *in situ*, which consisted of heating slowly the substrate up to the growth temperature so as to keep the pressure below 2 × 10^–7^ mbar and below 2 × 10^–9^ mbar before growth. The surface of the SrTiO_3_(001) substrate was always monitored before and during the film growth and showed a similar RHEED pattern across the sample series. The evaporation rates are determined for each material from a calibrated thickness monitor (2% error), while the La_0.8_Sr_0.2_MnO_3_ thickness was monitored in real time by following the RHEED intensity oscillations; for the buffer layer, it was not possible to determine the exact thickness exclusively on the basis of the RHEED oscillations since they are not observable at the early stages of deposition. The LaMnO_3_ and La_0.45_Sr_0.55_MnO_3_ thicknesses are hence estimated from the total growth time and the deposition rate, estimated by the RHEED oscillations appearing later in the deposition process. For this reason, we take into account an experimental error of 1 uc in the LaMnO_3_ thickness. In Figure 1a–c, we show representative RHEED patterns for the TiO_2_-terminated SrTiO_3_ substrate, LaMnO_3_, and La_0.8_Sr_0.2_MnO_3_ films after growth, respectively, demonstrating epitaxial growth and good surface properties. The observation of RHEED intensity oscillations, as illustrated in Figure 1d for the 3 uc La_0.8_Sr_0.2_MnO_3_/LaMnO_3_ film, indicates layer-by-layer growth; from atomic force microscopy (AFM) measurements, such as shown in Figure 1f, one observes the atomic steps from the substrate with a step height of ∼4 Å, i.e., of a single atomic unit cell, showing that the manganite films grow in single unit cell steps. After growth, the samples belonging to the LaMnO_3_ series are annealed in air at 600 °C for 6 h to fully oxygenate the films. Henceforth, the samples are named with the thickness of the top La_0.8_Sr_0.2_MnO_3_ layer in uc. The atomic force microscopy (AFM) measurements were performed in the tapping mode using a Bruker dimension icon 3100 instrument.

X-ray diffraction measurements were carried out in a Seifert four-circle diffractometer equipped with a Cu anode X-ray tube and a linear array detector. The measurements consisted of θ–2θ scans along the (002) diffraction peak to determine the out-of-plane lattice parameter and reciprocal space mapping scans around the (103) diffraction peak to extract the in-plane lattice parameter. The transport properties were measured in a home-built four-probe setup in a van der Pauw configuration,^37,38^ which uses spring contacts for a fast measurement setup; one drawback is that it leads to strong variations in the contact resistance, which we attribute to surface adsorbates due to the high vacuum or to surface residues from previous cleaning steps, making estimates of the absolute resistivity difficult. SQUID magnetometry measurements were performed using a 7 T Quantum Design MPMS3–137 magnetic properties measurement system (MPMS) with the magnetic field applied along the film plane. X-ray absorption spectroscopy (XAS) measurements were carried out at the SIM and X-Treme beamlines at the Swiss Light Source (SLS) at the Paul Scherrer Institute (PSI), Switzerland, with the signal collected in the total electron yield (TEY) mode, which has a high surface sensitivity due to the limited mean free path of the electrons in solid materials on the order of 3 nm, or about 8 uc, and the exponential attenuation of the TEY signal with thickness. When measuring at SIM, before each magnetic XAS measurement, a saturation field of 1 kOe was applied to the sample and the measurements were performed under a 200 Oe magnetic field. At X-Treme, the saturation field was 6 T and the measurements were performed at remanence. We collected XAS spectra with circularly left and right polarized light, from which we obtained the unpolarized (average) spectra and the X-ray magnetic circular dichroism (XMCD) spectra (difference). The latter are normalized by dividing it by the L_3_ peak intensity of the respective average XAS spectrum to give the dichroic signal as a percentage of the average XAS spectrum. Linear dichroism measurements were carried out by measuring the XAS spectra with the sample at a normal angle of incidence and at 45° to the X-ray beam to probe, respectively, the in-plane and out-of-plane orbital states; for one sample (5 uc La_0.8_Sr_0.2_MnO_3_/La_0.45_Sr_0.55_MnO_3_), the X-ray linear dichroic (XLD) measurements were carried out using the X-ray photoemission electron microscope at the SIM beamline, where the light impinges the sample at a grazing angle of 16°. The different experimental conditions for these measurements (i.e., different monochromator grating and beam slit settings) result in a lower energy resolution of the 5 uc spectra. In order to compare spectra taken at different geometries, the out-of-plane XAS spectra are calculated using the expression for the angular dependence of the linear dichroism, *I*(θ) = *I*_ab_ cos^2^ θ + *I*_c_ sin^2^ θ, where θ is the angle of incidence of the light with respect to the sample surface and *I*_ab_ and *I*_c_ are the X-ray absorption parallel to the sample surface and perpendicular to it, respectively.

## 3 Results and Discussion

**Figure 2:**
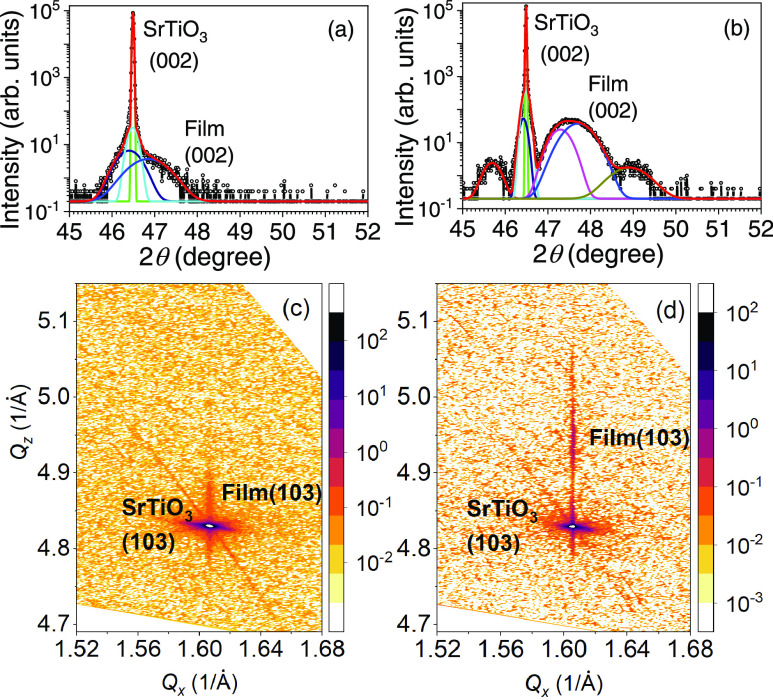
θ–2θ X-ray diffraction scans for (a) 10 uc La_0.8_Sr_0.2_MnO_3_/LaMnO_3_ and (b) 10 uc La_0.8_Sr_0.2_MnO_3_/La_0.45_Sr_0.55_MnO_3_ heterostructures. Reciprocal space mapping around the asymmetric (103) Bragg reflection of SrTiO_3_ for (c) 10 uc La_0.8_Sr_0.2_MnO_3_/LaMnO_3_ and (d) 10 uc La_0.8_Sr_0.2_MnO_3_/La_0.45_Sr_0.55_MnO_3_. The vertical scale represents the scattering intensity in arbitrary units.

To determine the strain state of the heterostructures, we carried out X-ray diffraction measurements on the two 10 uc La_0.8_Sr_0.2_MnO_3_ films. The θ–2θ scans around the (002) SrTiO_3_ peak are shown in Figure 2a,b for the LaMnO_3_ and La_0.45_Sr_0.55_MnO_3_ buffer layers, respectively. In both cases, broad peaks to the right of the (002) SrTiO_3_ line are observed, corresponding to the pseudocubic (002) planes of the manganite films; in the case of the LaMnO_3_ buffer layer, only one peak can be distinguished. By fitting the peak with three Gaussian components (Figure 2a), one for the SrTiO_3_ peak together with a diffused scattering component and a third for the LaMnO_3_/La_0.8_Sr_0.2_MnO_3_ bilayer, we obtain for the latter an out-of-plane lattice parameter of 3.87 Å, which is below both LaMnO_3_ and La_0.8_Sr_0.2_MnO_3_ pseudocubic bulk values of *a*/√2 = 3.89 Å.^13^ For the case of the film with the La_0.45_Sr_0.55_MnO_3_ buffer layer, we observe the presence of clear Laue oscillations, indicative of high-quality interfaces and that the manganite peak is composed of two overlapping components (Figure 2b), which we ascribe to the La_0.8_Sr_0.2_MnO_3_ and La_0.45_Sr_0.55_MnO_3_ films. By fitting this peak to two Gaussian components together with the SrTiO_3_ peak with a diffused scattering component, we obtain out-of-plane lattice parameters of 3.84 and 3.81 Å, which we assign to the La_0.8_Sr_0.2_MnO_3_ and La_0.45_Sr_0.55_MnO_3_ films, respectively. These values are considerably smaller than the respective pseudocubic bulk lattice parameters of 3.89 and 3.84 Å,^13,34,39^ as expected for the tensile strain induced by the SrTiO_3_ substrate.

Reciprocal space mapping (RSM) around the asymmetric (103) plane of SrTiO_3_ is shown in Figure 2c,d for La_0.8_Sr_0.2_MnO_3_/LaMnO_3_ and La_0.8_Sr_0.2_MnO_3_/La_0.45_Sr_0.55_MnO_3_, respectively. One finds that the Bragg reflection corresponding to the film bilayer lies at the same *Q*_*x*_ value for both heterostructures, which directly shows that the manganite films grow coherently to the SrTiO_3_ substrate and are fully strained. We expect likewise the thinner La_0.8_Sr_0.2_MnO_3_ films to be also fully strained to the SrTiO_3_ substrate.

**Figure 3:**
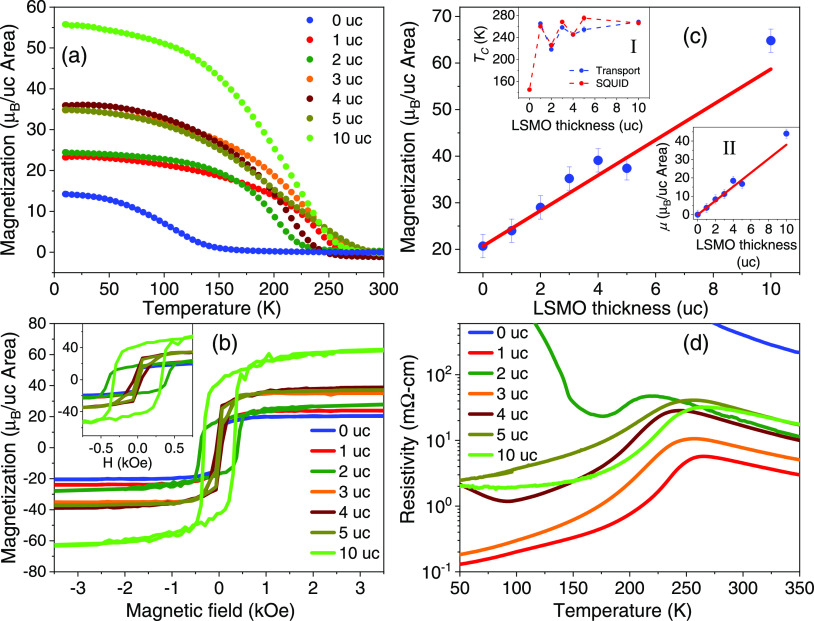
(a) Field-cooled magnetization variation with temperature of the La_0.8_Sr_0.2_MnO_3_/LaMnO_3_ films taken during heating (applied magnetic field of 1 kOe). (b) Magnetic hysteresis curves at 20 K. Inset: Zoomed-in *M*–*H* curves highlighting the low field magnetic behavior. (c) Zero-field saturation magnetization per unit cell area versus La_0.8_Sr_0.2_MnO_3_ film thickness (symbols) extrapolated from the *M*-*H* loops; the red line has a slope of 3.8 μ_B_/uc. Inset (I) shows the variation of the critical temperature (SQUID) and of the peak in resistivity as a function of La_0.8_Sr_0.2_MnO_3_ film thickness (dashed lines are guides to the eye); inset (II) shows the magnetic moment (μ) variation without the LaMnO_3_ moment contribution. The error bars in the magnetization are related to the experimental uncertainty due to the LaMnO_3_ thickness. (d) Resistivity versus temperature for the La_0.8_Sr_0.2_MnO_3_/LaMnO_3_ sample series.

The temperature dependence of the magnetization (*M*–*T*) of the ultrathin La_0.8_Sr_0.2_MnO_3_/LaMnO_3_ films as a function of La_0.8_Sr_0.2_MnO_3_ thickness is shown in Figure 3a. A first observation is that the 12 uc LaMnO_3_/SrTiO_3_ (001) film exhibits a nonzero saturation magnetization, which is not expected for stoichiometric bulk LaMnO_3_ but is similar to what has been reported previously in the literature for LaMnO_3_ thin films.^40−47^ The Curie temperature, *T*_C_ = 145 K, is coincidentally very close to the Néel temperature of bulk LaMnO_3_, of 143 K.^13^ While the origin of magnetism in LaMnO_3_ is still under debate, recent surface-sensitive X-ray photoemission spectroscopy measurements indicate the presence of Sr and Ca in the LaMnO_3_ film in both the as-grown and annealed states of up to a few %, suggesting that the magnetic moment in LaMnO_3_ may be driven in large part by doping from divalent cations diffusing from the substrate.^48^ For the La_0.8_Sr_0.2_MnO_3_ films, the results show that starting from 1 uc La_0.8_Sr_0.2_MnO_3_, the Curie temperature jumps to *T*_C_ = 260 K (compared with 309 K for bulk La_0.8_Sr_0.2_MnO_3_ and 305 K for thick La_0.8_Sr_0.2_MnO_3_/STO(001), slightly reduced from the bulk value due to epitaxial tensile strain);^13,17^ with increasing La_0.8_Sr_0.2_MnO_3_ thickness, *T*_C_ is seen to oscillate around 250 K, as shown in inset I of Figure 3c, which could be a manifestation of finite-size effects.^3^ Notably, we find no independent contribution from the LaMnO_3_ buffer layer in the *M*–*T* curves, indicating that the two layers are fully coupled magnetically. We also observe a steady increase in the saturation magnetization with increasing thickness, which we quantify from magnetic hysteresis curves carried out at 20 K, shown in Figure 3b. These data show that the La_0.8_Sr_0.2_MnO_3_ films have small coercive fields, of ∼50 Oe, except for the 2 and 10 uc La_0.8_Sr_0.2_MnO_3_ films, which exhibit coercive fields of ∼300 Oe. We attribute the larger coercive field to sample-to-sample growth-related variations. The larger coercivity implies stronger energy barriers to domain wall motion or to nucleation of reverse domains; given the high sensitivity of the coercive field to extrinsic factors, including local structural variations induced by the substrate morphology (such as degree of miscut, surface roughness, and point defects), some variation in the coercive field may be expected. By extrapolating from the high field region to zero field, we estimate the zero-field saturation magnetization as a function of thickness, shown in Figure 3c, to find that the saturation moment increases linearly with thickness, with a slope of 3.8 μ_B_/uc expected for bulk La_0.8_Sr_0.2_MnO_3_, shown as a red line. This result demonstrates that the insertion of a LaMnO_3_ buffer layer results in La_0.8_Sr_0.2_MnO_3_ films that are fully magnetically polarized down to 1 uc. The intercept at the origin gives the magnetization of LaMnO_3_, of 20.7 μ_B_/uc area, corresponding to an average magnetic moment of 1.7 μ_B_/Mn, in agreement with previous findings in the literature.^42,47^ Assuming the LaMnO_3_ moment to be constant throughout the sample series, we can subtract its (constant) magnetic contribution to obtain the linear behavior shown in inset II of Figure 3c. For the 3 uc La_0.8_Sr_0.2_MnO_3_ thickness, we expect a slightly larger magnetic moment due to the thicker LaMnO_3_ layer (14 uc), and in inset II of Figure 3c, we show the magnetic moment after subtracting an additional moment of 3.4 μ_B_ corresponding to the extra 2 uc LaMnO_3_. These results highlight the fact that the La_0.8_Sr_0.2_MnO_3_ films develop a full magnetic moment starting from the first unit cell. In our discussion, we have supposed that no interdiffusion between the two layers occurs, i.e., that the two layers are well-defined. This is supported by our results: a systematic increase in the saturation magnetization as a function of the top La_0.8_Sr_0.2_MnO_3_ thickness of 3.8 μ_B_/uc indicates that interdiffusion, if present, is not the major factor driving the observed phenomena.

The transport properties are listed in Figure 3d. A first remark is that the relative variation of the resistivity with La_0.8_Sr_0.2_MnO_3_ film thickness does not follow the expected behavior, namely, a decrease with increasing film thickness. We attribute this largely to contact resistance introduced by the spring contacts in our measurement setup; coincidentally, the 2 and 10 uc La_0.8_Sr_0.2_MnO_3_ films showing a larger coercive field also have a systematically larger resistivity, a property which is also very sensitive to local morphological and structural variations. However, while the presence of defects in the samples is expected to impact the resistivity and coercive field, we find that it does not strongly affect the saturation moments, as the *M*–*H* curves indicate. From the temperature dependence, we find that the 12 uc thick LaMnO_3_ film is insulating, consistent with previous reports and as expected for bulk LaMnO_3_;^13,33^ importantly, starting from 1 uc thickness, the films exhibit a clear peak in the resistivity at a temperature near the magnetic critical temperature (inset I of Figure 3c), typical of the mixed-valence “colossal” magnetoresistance (CMR) manganites.^49^ The 2 and 4 uc La_0.8_Sr_0.2_MnO_3_ films show a second transition to insulating behavior at low temperatures, which is often found in thin La_0.8_Sr_0.2_MnO_3_/SrTiO_3_(001) films and attributed to A-site disorder-induced charge localization effects.^50^ These results show that also the transport properties of the ultrathin La_0.8_Sr_0.2_MnO_3_ films are similar to that of the bulk counterpart.^13^ The La_0.8_Sr_0.2_MnO_3_ films are conducting and exhibit a metal-to-insulator transition starting from 1 uc, which demonstrates that the La_0.8_Sr_0.2_MnO_3_ is in a metallic ferromagnetic state and has no electric dead layer as is the case with La_1–*x*_Sr_*x*_MnO_3_ films grown on SrTiO_3_, for example, and that the peak in resistivity agrees with *T*_c_ obtained from the *M*–*T* curves, which is another characteristic of the mixed-valence manganites. These are important aspects for utilizing such ultrathin films for field effect devices or as polarizers in tunnel barriers.

**Figure 4:**
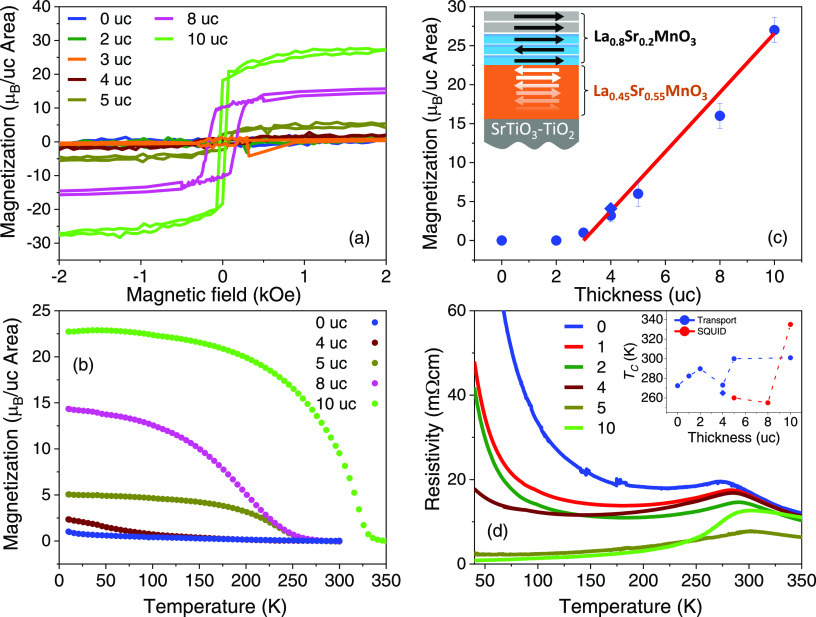
(a) Magnetization hysteresis curves of the La_0.8_Sr_0.2_MnO_3_/La_0.45_Sr_0.55_MnO_3_ sample series (20 K) and (b) magnetization temperature dependence of the 0, 4, 5, 8, and 10 uc La_0.8_Sr_0.2_MnO_3_ films (1 kOe). (c) Zero-field saturation moments extrapolated from the *M*–*H* loops. The red line has a slope of 3.8 μ_B_/uc crossing the abscissa at 3 uc. Inset: Schematic representation of the magnetic spin configuration of the La_0.8_Sr_0.2_MnO_3_ film on the La_0.45_Sr_0.55_MnO_3_ buffer layer. (d) Transport measurements showing the evolution of the temperature-dependent resistivity with thickness. The metal-to-insulator transition variation with thickness, identified as *T*_C_, is shown in the figure inset together with the Curie temperature, obtained as a linear extrapolation to zero from the *M*–*T* curves. The diamond symbols in (c) and (d) are from a second 4 uc La_0.8_Sr_0.2_MnO_3_/La_0.45_Sr_0.55_MnO_3_ sample grown subsequently.

The results for the case of La_0.8_Sr_0.2_MnO_3_/La_0.45_Sr_0.55_MnO_3_ are markedly different. The magnetic hysteresis loops, presented in Figure 4a, show that the La_0.45_Sr_0.55_MnO_3_/SrTiO_3_(001) film exhibits no magnetic hysteresis, consistent with the expected antiferromagnetic state reported earlier.^34^ Also, the La_0.8_Sr_0.2_MnO_3_ films up to 3 uc thickness show a negligible magnetic response to the applied magnetic field, while for thicknesses above 4 uc, magnetic hysteresis is present with coercive fields in the range from 50 to 100 Oe. The magnetic behavior is also confirmed by temperature-dependent magnetization measurements shown in Figure 4b for the 4, 5, 8, and 10 uc films, where the onset of magnetization in the system is observed at 250 K (with the 4 uc film showing an uptick in the magnetization at around 100 K; this behavior was reproduced in a second sample grown subsequently). Remarkably, the 10 uc La_0.8_Sr_0.2_MnO_3_ film reaches a critical temperature of about 300 K, close to the bulk value. The saturation magnetic moment extracted from the *M*–*H* curves is presented in Figure 4c and shows an approximately linear increase in magnetization for thicknesses above 3 uc with a slope of about 3.8 μ_B_/uc.

The resistivity of the La_0.8_Sr_0.2_MnO_3_ films grown on La_0.45_Sr_0.55_MnO_3_ as a function of temperature is shown in Figure 4d. Since in this case the buffer layer brings a non-negligible contribution to the total resistivity, the latter is calculated using the bilayer film thickness. The 0 uc sample shows a metal-to-insulator transition (MIT) at *T*_MIT_ = 287 K, in agreement with a previous report.^34^ The reduced resistivity and increased MIT temperature (*T*_MIT_) of the 1, 2, and 3 uc samples show that the La_0.8_Sr_0.2_MnO_3_ layers have a lower resistivity compared to the buffer layer. The 4 uc La_0.8_Sr_0.2_MnO_3_/La_0.45_Sr_0.55_MnO_3_ shows instead a drop in *T*_MIT_, as also shown in the figure inset (we confirmed this behavior from a second 4 uc La_0.8_Sr_0.2_MnO_3_/La_0.45_Sr_0.55_MnO_3_ sample). A further increase in *T*_MIT_ is observed for the 5 and 10 uc samples, saturating at ∼300 K. The critical temperature estimated from the peak resistivity is found to be significantly different from that estimated from the *M*–*T* curves, a discrepancy that is much larger than that for the La_0.8_Sr_0.2_MnO_3_/LaMnO_3_ series. For example, for the 5 uc film, the critical temperature obtained from the magnetization curve is about 260 K, while the peak in resistivity remains close to that of 3 uc La_0.8_Sr_0.2_MnO_3_, of about 290 K. Differently from the previous case, where the LaMnO_3_ buffer layer is insulating and *T*_MIT_ is entirely determined by the La_0.8_Sr_0.2_MnO_3_ layer itself, the La_0.45_Sr_0.55_MnO_3_ buffer layer is conducting; hence, the transport properties are determined by the interplay of the two layers. Therefore, although a formal distinction of the two layers cannot be done from the resistivity data, we can draw some conclusions by combining the transport and magnetization data: up to 4 uc, La_0.8_Sr_0.2_MnO_3_ couples antiferromagnetically to La_0.45_Sr_0.55_MnO_3_, which is inferred from the SQUID measurements and the systematic increase of *T*_MIT_ within this thickness range. When La_0.8_Sr_0.2_MnO_3_ becomes ferromagnetic, *T*_MIT_ is given by a weighted average of the two contributions corresponding to the Curie (for La_0.8_Sr_0.2_MnO_3_) and Néel (for La_0.45_Sr_0.55_MnO_3_) temperatures. The fact that they do not agree suggests that at and above 5 uc thickness, the ferromagnetic component of the La_0.8_Sr_0.2_MnO_3_ film is magnetically decoupled from the manganite layers underneath. An alternative explanation, given that one does not clearly distinguish two peaks in the resistivity, could be that the top La_0.8_Sr_0.2_MnO_3_ films switch from an antiferromagnetic state at higher temperatures to the ferromagnetic ground state with decreasing temperatures. The kink at around 100 K observed for the 1–4 uc films is due to the SrTiO_3_ cubic-to-tetragonal phase transition, which affects particularly strongly La_1–*x*_Sr_*x*_MnO_3_ films at near the 0.5 doping.^34,51^

We interpret the results for La_0.8_Sr_0.2_MnO_3_/La_0.45_Sr_0.55_MnO_3_ as showing that the 1–3 uc La_0.8_Sr_0.2_MnO_3_ films either are antiferromagnetic or couple antiferromagnetically to the La_0.45_Sr_0.55_MnO_3_ buffer layer with the possible presence of antiferromagnetic domains in the La_0.45_Sr_0.55_MnO_3_ buffer layer averaging out the net magnetization, while for larger thicknesses, the additional layer changes abruptly to a ferromagnetic state. X-ray photoemission electron microscopy results show, in fact, that for the 1–3 uc La_0.8_Sr_0.2_MnO_3_ film no ferromagnetic contrast over an antiferromagnetic multidomain state is present, consistent with the scenario that at small thicknesses, the thinner La_0.8_Sr_0.2_MnO_3_ films order antiferromagnetically.^52^ The increase in the Néel temperature (corresponding to *T*_MIT_ in these films) for 1–3 uc indicates an increase of the magnetic exchange interaction, while the large difference between *T*_C_ and *T*_MIT_ shows that the ferromagnetic layers are magnetically decoupled from the antiferromagnetic layer underneath. Starting from 4 uc thickness, the magnetic moment increases linearly with a slope of 3.8 μ_B_/Mn, indicated by the red line in Figure 4c, similar to the LaMnO_3_ buffer layer case but shifted by 3 uc. We mention here the possibility for frustration at the interface, which was found to be present in La_1–*x*_Sr_*x*_MnO_3_/La_0.6_Sr_0.4_FeO_3_ multilayers, where La_0.6_Sr_0.4_FeO_3_ is an antiferromagnetic system displaying a C-type canted antiferromagnetic spin state that leads to spin frustration at the La_1–*x*_Sr_*x*_MnO_3_ interface and to more complex spin configurations.^53^ In our situation, we expect La_0.45_Sr_0.55_MnO_3_ to be in an A-type antiferromagnetic state, consisting of ferromagnetic spin planes coupled antiferromagnetically along the [001] direction. Such a magnetic spin configuration should not lead to spin frustration at the La_0.8_Sr_0.2_MnO_3_ interface.

**Figure 5:**
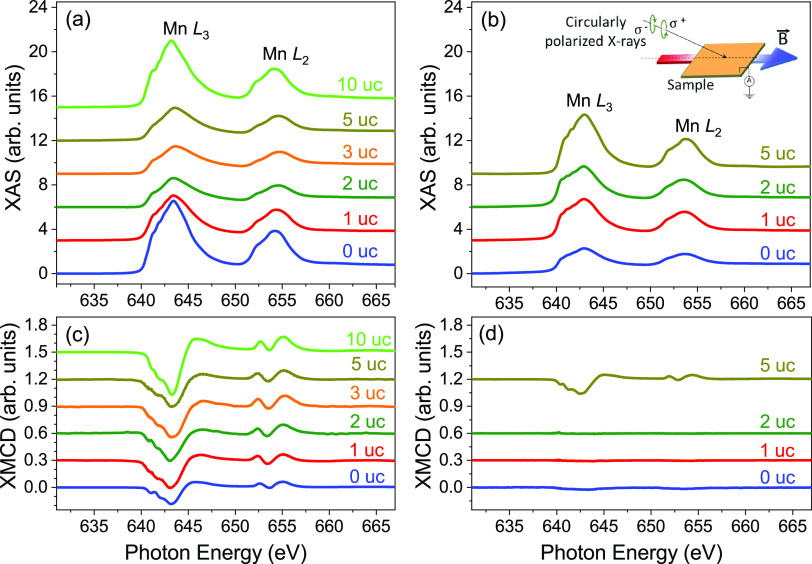
XAS spectra collected on La_0.8_Sr_0.2_MnO_3_ films grown on (a) the LaMnO_3_ and (b) the La_0.45_Sr_0.55_MnO_3_ buffer layer. The corresponding XMCD spectra, normalized to the respective XAS peak value, are shown in (c) and (d). All spectra are shifted vertically for clarity of display.

To better understand the magnetic and electronic properties of the La_0.8_Sr_0.2_MnO_3_ films, we carried out X-ray magnetic spectroscopy measurements. In Figure 5a,b, we show the evolution of the nonpolarized XAS spectra with thickness for the films grown on the LaMnO_3_ and La_0.45_Sr_0.55_MnO_3_ buffer layers, respectively. For the case of the films grown on LaMnO_3_, we find no significant changes in the spectra across the sample series, which show features characteristic of fully oxidized La_0.8_Sr_0.2_MnO_3_.^54,55^ (The spectra corresponding to the 0 and 10 uc samples were collected at the X-Treme beamline and show a different peak amplitude, attributed to different background and measurement conditions between the two endstations; we aligned the energy scale between these two sets of measurements by matching the peak energy of the 10 uc film to that of the 5 uc). Although one may expect a small shift in the peak energy position when going from LaMnO_3_ to La_0.8_Sr_0.2_MnO_3_, the shift is relatively small,^56^ compounded by the fact that the LaMnO_3_ film is itself slightly doped. The corresponding normalized XMCD spectra are shown in Figure 5c. Similar to XAS, we find no significant changes in the shape and amplitude of the XMCD spectra; the 0 and 10 uc samples show a reduced and an enhanced XMCD signal, respectively. These results confirm that the magnetic moment originates from the Mn cations and are in agreement with the bulk magnetometry results showing full magnetic moment of the La_0.8_Sr_0.2_MnO_3_ film: since the spectra are collected in TEY, which has a sensitivity of around 3–5 nm due to the limited electron mean free path, and is therefore representative of the magnetic state of the surface layers,^57^ a constant XMCD signal with thickness confirms that each added layer is magnetic. Interestingly, since the amplitude of the LaMnO_3_ buffer layer XMCD is comparable to that of the other samples in the series and the XMCD amplitudes are normalized to the XAS spectra, our results suggest that the magnetic moment in LaMnO_3_ arises from the surface layer. The La_0.45_Sr_0.55_MnO_3_ sample series was measured at the SIM beamline, with the exception of the 5 uc, measured at X-Treme. The shape of the main peak at 642 eV is an indication that the films are stoichiometric and fully oxidized, with Mn present in a mixed 3+/4+ valence state.^56^Figure 5d shows the corresponding XMCD spectra. Consistent with the magnetometry results, the 0, 1, and 2 uc samples show no XMCD signal (the residual signal for 0 uc results from a slight difference in amplitude for the circular right and left light that could not be corrected in the data and does not have the expected XMCD energy variation), while the 5 uc sample displays a strong magnetic dichroic response, but with an amplitude that is lower than the case of the LaMnO_3_ series. These results support the conclusion that the magnetic signal arises from the top two unit cells and is slightly reduced by the zero XMCD signal contribution from the antiferromagnetic La_0.8_Sr_0.2_MnO_3_/La_0.45_Sr_0.55_MnO_3_ films underneath.

**Figure 6:**
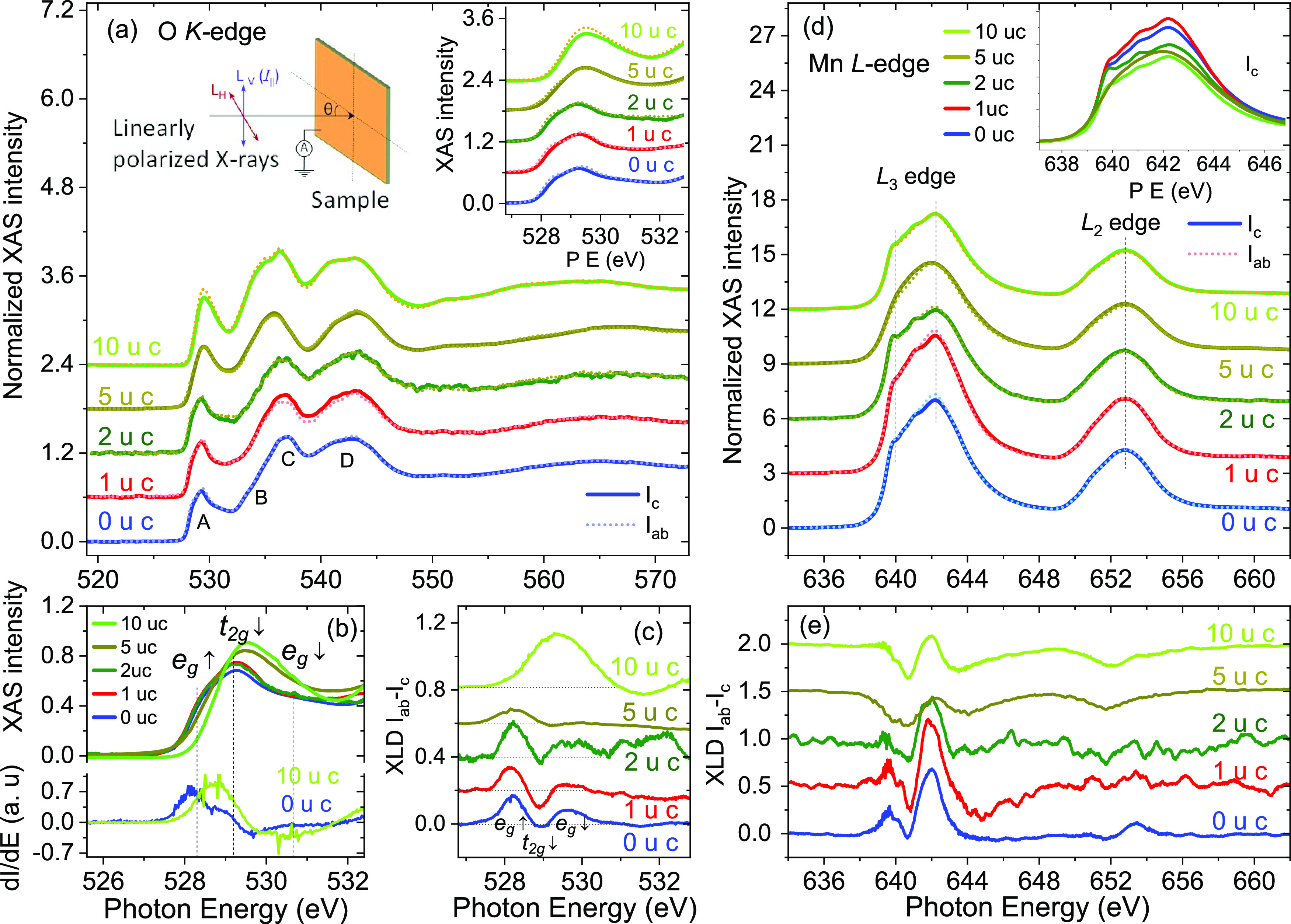
(a) Room-temperature XAS spectra at the O K-edge obtained with linearly polarized light for La_0.8_Sr_0.2_MnO_3_/La_0.45_Sr_0.55_MnO_3_. (b) Detail of the pre-edge feature of the spectra (out-of-plane light polarization) highlighting the transition associated with the O 2p-Mn 3d hybridized states. The derivatives of the 1 and 10 uc spectra are also shown to highlight the peak position. (c) O K-edge XLD spectra of the La_0.8_Sr_0.2_MnO_3_ films. (d) XAS spectra at the Mn L-edge collected with vertical (*I*_ab_) and horizontal (*I*_c_) light polarization. Inset shows a plot of all spectra at the L_3_-edge. (e) Mn L-edge XLD spectra. Spectra are shifted vertically for clarity of display.

Magnetic exchange in these materials is generally understood in terms of orbital occupancy, according to the Goodenough–Kanamori rules.^12,58^ In particular, doping and strain play fundamental roles in determining which of the spin exchange mechanisms, double exchange or superexchange, dominates the magnetic interaction since the orbital structure depends strongly on those parameters. In order to probe the orbital character of the La_0.8_Sr_0.2_MnO_3_/La_0.45_Sr_0.55_MnO_3_ heterostructures, we carried out X-ray absorption measurements with linearly polarized light, which probes the available density of states along the electric field direction.^59^ The XAS spectra measured at 300 K, above the Curie temperature to avoid the magnetic contribution, are shown in Figure 6a,d for the O K-edge and Mn L-edge, respectively.

The O K-edge shows the general shape typical for La_0.8_Sr_0.2_MnO_3_, with pre-edge peaks (A, B) associated with O 2p orbitals hybridized with Mn 3d and higher energy peaks, C, D, associated with O 2p orbitals hybridized with La and Sr orbitals, respectively.^56^ Of interest here are the pre-edge peaks, in the range 527–533 eV, which are argued to reflect more directly the unoccupied density of states of the Mn 3d states, since the excitation occurs at the oxygen site.^60^ Two different assignments of these peaks to the Mn orbitals states have been presented in the literature, in one case where peak A is assigned to the partially filled *e*_*g*_^↑^ band and peak B to transitions into empty *t*_2*g*_^↓^ and *e*_*g*_^↓^ states;^56,61,62^ another assignment ascribes peak A to the partially filled *e*_*g*_^↑^ band and empty *t*_2*g*_^↓^ and peak B to transitions into *e*_*g*_^↓^ states.^63^ Our results are not able to address this controversy; however, we can extract the following conclusions from our data.

(i) One finds a shift to higher energies of the O pre-edge features associated with the *e*_*g*_^↑^ band when going from 0, 1, and 2 uc La_0.45_Sr_0.55_MnO_3_ to 5 and 10 uc La_0.8_Sr_0.2_MnO_3_ (Figure 6b); this is expected based on band filling, since with decreasing hole doping the electron occupation of the *e*_*g*_^↑^ band increases, resulting in a decrease in the unoccupied density of states.
(ii) One finds that the leading edge of the X-ray absorption spectra occurs at lower energies for the in-plane polarized light, while the difference is significantly reduced for the 5 and 10 uc films; this can be more easily seen as a shift to higher energies of the spectral weight of the linear dichroic signal at the *e*_*g*_^↑^ band, Figure 6c. This behavior indicates a reduction in the asymmetry of the occupancy of in-plane and out-of-plane orbitals at larger La_0.8_Sr_0.2_MnO_3_ thicknesses, consistent with what is expected for the ferromagnetic state.^64^ Furthermore, the role of strain is that of shifting the relative energy position of the two *e*_*g*_^↑^ orbitals, *z*^2^ and *x*^2^ – *y*^2^, with compressive (negative) strain favoring the *x*^2^ – *y*^2^ orbitals.^65^ We note that, given that both layers have the same in-plane lattice parameter and given that the lattice parameter of La_1–*x*_Sr_*x*_MnO_3_ changes with Sr doping, the La_0.45_Sr_0.55_MnO_3_ buffer layer and the La_0.8_Sr_0.2_MnO_3_ top layer are subjected to different in-plane strains. Taking the pseudocubic lattice parameters for La_0.45_Sr_0.55_MnO_3_ and La_0.8_Sr_0.2_MnO_3_ as 3.84 and 3.89 Å, respectively,^13,34,39^ one obtains in-plane strains of −1.69 and −0.39%, respectively. Thus, our results agree with the expectations and are consistent with the magnetic behavior, i.e., an antiferromagnetic in-plane metallic A-type state for La_0.45_Sr_0.55_MnO_3_ and a ferromagnetic isotropic state for La_0.8_Sr_0.2_MnO_3_.
(iii) In terms of the thickness evolution, one finds that up to 2 uc La_0.8_Sr_0.2_MnO_3_ the XLD spectra in Figure 6e remain essentially unchanged (although in these cases, a significant signal contribution from the underlying La_0.45_Sr_0.55_MnO_3_ layer should be present), while for 5 uc, which is expected to have a strongly reduced La_0.45_Sr_0.55_MnO_3_ signal contribution due to the limited electron escape depth, a strong modification in the XAS spectra is observed, starting to resemble the 10 uc La_0.8_Sr_0.2_MnO_3_ spectra.
(iv) The integral of the linear dichroism over the Mn L_2,3_ edge, Figure 6e, gives the preferred hole orbital occupation according to the linear dichroism sum rule *D*_L_ = ∫_L_2_+L_3__(μ_ab_ – μ_c_) d*E*/∫_L_2_+L_3__(2μ_ab_ + μ_c_)d*E* ∝ ∑⟨*l*_*z*_^2^ – 2⟩_*i*_, where μ_ab_ and μ_c_ are the X-ray absorption along the in-plane and out-of-plane directions, respectively, *l*_*z*_ is the quantum orbital number, with *l*_*z*_ = 0 for the 3d_3*z*^2^–*r*^2^_ orbital and *l*_*z*_ = 2 for 3d_*x*^2^–*y*^2^_.^66^ A positive value for *D*_L_ indicates a preferential hole occupation of the *x*^2^ – *y*^2^ orbitals. Based on this sum rule, one finds, qualitatively, that for 0–2 uc La_0.8_Sr_0.2_MnO_3_ the in-plane *x*^2^ – *y*^2^ orbitals are preferentially occupied by hole carriers,^61,67^ while for 5 and 10 uc, there is a change in the orbital character to a predominant hole occupation of the out-of-plane orbitals. The changes in the orbital character are in general qualitative agreement with the O pre-edge spectral features.

**Figure 7:**
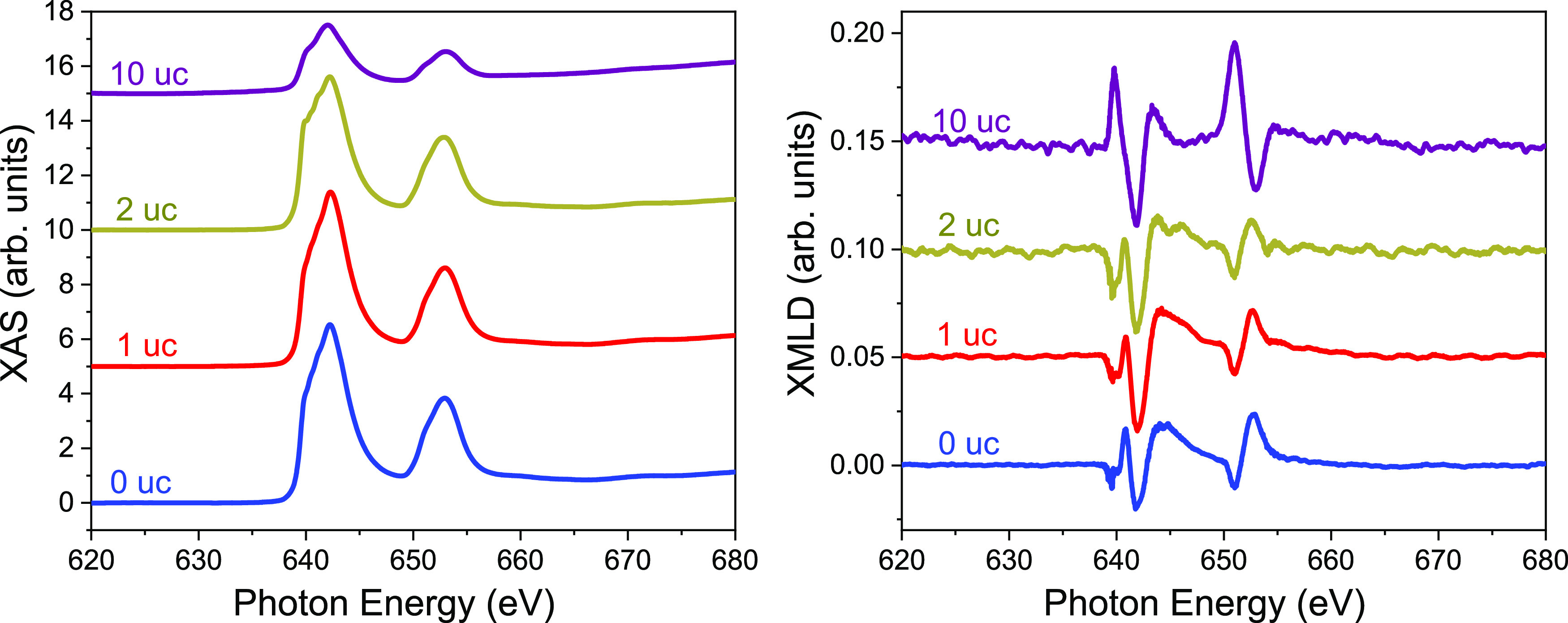
Average XAS (left) and XMLD (right) scans for samples 0, 1, 2, and 10 uc La_0.8_Sr_0.2_MnO_3_/La_0.45_Sr_0.55_MnO_3_ taken at 20 K. The XMLD spectra were normalized to the XAS maximum value. Spectra are shifted vertically for clarity of display.

We also carried out magnetic linear dichroism (XMLD) measurements for 0, 1, 2, and 10 uc La_0.8_Sr_0.2_MnO_3_/La_0.45_Sr_0.55_MnO_3_ at 20 K, as shown in Figure 7. The XMLD signal is normalized to the XAS peak intensity, and these measurements are taken with a large beam spot (of about 1 mm in lateral size), averaging over a large area of the sample, i.e., it is an average over the antiferromagnetic multidomain state of the sample. Assuming that for all samples there is a similar antiferromagnetic domain distribution, these data tell us that the amplitude of the XMLD signal remains approximately constant with increasing film thickness, which indicates that each added layer contributes similarly to the XMLD signal (otherwise, the relative magnetic contribution to the total signal would drop). These data further support our conclusion that the top La_0.8_Sr_0.2_MnO_3_ layer is in an antiferromagnetic state at lower thicknesses, as discussed above.

From these observations, in combination with the magnetic behavior, we conclude that La_0.8_Sr_0.2_MnO_3_ films up to 3 uc thickness have a similar electronic and magnetic structure as the La_0.45_Sr_0.55_MnO_3_ buffer layer, characterized by a preferential hole occupation of the *x*^2^ – *y*^2^ orbitals, large in-plane conductivity, and an A-type antiferromagnetic ordering. At larger thicknesses, a transition to a preferential occupation of out-of-plane orbitals occurs, where a spectrum similar to that reported for the fully polarized ferromagnetic state is observed,^68^ even if an equal population of both in-plane and out-of-plane orbitals were expected.^69^

The two different buffer layers, therefore, lead to two distinct magnetic behaviors for the top La_0.8_Sr_0.2_MnO_3_ films. In the case of LaMnO_3_, the La_0.8_Sr_0.2_MnO_3_ films are fully magnetically polarized starting from 1 uc, with a magnetic critical temperature that is much higher than that of the single buffer layer, indicating the presence of a strong magnetic coupling dominated by the top La_0.8_Sr_0.2_MnO_3_. Since the LaMnO_3_ film is insulating, we attribute the magnetic coupling to be dominated by double exchange at the interface between the two systems. In the case of the La_0.45_Sr_0.55_MnO_3_ buffer layer, we find that the La_0.8_Sr_0.2_MnO_3_ film adopts the metallic antiferromagnetic state of the buffer layer up to a thickness of 3–4 uc and a sudden transition to a ferromagnetic state above this thickness, manifested in the linear dichroic data by a sudden modification of the electronic structure from 2 to 5 uc films. We explain the interfacial antiferromagnetic metallic state as driven by extended delocalized charge transfer between La_0.45_Sr_0.55_MnO_3_ and La_0.8_Sr_0.2_MnO_3_, which operates up to three unit cells beyond the interface. At larger thicknesses, the La_0.8_Sr_0.2_MnO_3_ adopts its ferromagnetic ground state with a full bulk moment and critical temperature independent of the underlying manganite film. In both instances, the La_0.8_Sr_0.2_MnO_3_ films are fully magnetically polarized, starting from the single unit cell. One possible common mechanism driving the magnetic behavior of the top La_0.8_Sr_0.2_MnO_3_ films toward bulk behavior may be the onset of oxygen octahedral rotations promoted by the LaMnO_3_ buffer layer and the first 3–4 unit cells La_0.8_Sr_0.2_MnO_3_/La_0.45_Sr_0.55_MnO_3_, which agrees with the length scale for bond angle relaxation of the oxygen octahedral tilt of 4 unit cells found for LaMnO_3_/SrTiO_3_ superlattices.^70^

Our results are highly relevant for device applications since they provide an approach to using the same material platform to create La_0.8_Sr_0.2_MnO_3_ films that are fully magnetically polarized down to 1 uc either on conducting buffer layers (La_0.45_Sr_0.55_MnO_3_) or on magnetic but insulating layers (LaMnO_3_). We anticipate as well that the results found here may be applicable to the wider doping range where La_1–*x*_Sr_*x*_MnO_3_ is in a ferromagnetic state, including optimally doped La_1–*x*_Sr_*x*_MnO_3_, and to the wider manganite perovskite family.

## 4 Conclusions

In conclusion, we have studied the evolution of the magnetic moment of ultrathin La_0.8_Sr_0.2_MnO_3_ films grown on insulating LaMnO_3_ and conducting La_0.45_Sr_0.55_MnO_3_ buffer layers. Although both systems are nominally antiferromagnetic, we find a significant magnetic moment in the LaMnO_3_ buffer layer, a result similar to that reported previously in the literature. For La_0.8_Sr_0.2_MnO_3_/LaMnO_3_, a metallic state and bulk-like magnetic moment are observed for La_0.8_Sr_0.2_MnO_3_ thicknesses down to 1 uc. The two layers are magnetically coupled, but the magnetic properties are dominated by the top La_0.8_Sr_0.2_MnO_3_ film, including bulk-like magnetic moments and high critical temperatures that do not correspond to those of the bare LaMnO_3_ film. For the case of La_0.8_Sr_0.2_MnO_3_/La_0.45_Sr_0.55_MnO_3_, we observe an antiferromagnetic ground state up to 3 uc, above which a ferromagnetic ground state for the subsequent La_0.8_Sr_0.2_MnO_3_ layers emerges. The X-ray absorption spectroscopy results show a sudden change in the electronic structure from 2 to 5 uc, consistent with the observed change in the magnetic properties. Our results show that the properties of ultrathin La_0.8_Sr_0.2_MnO_3_ films can be tailored by a suitable choice of buffer layers to yield bulk-like magnetic polarization and high critical temperatures, down to the unit cell thickness range, paving the way to fully exploring the unique electronic properties of this class of strongly correlated oxide materials.

## Data Availability

The data underlying this study are openly available at the PSI Public Data Repository database at doi.psi.ch/detail/10.16907/b927e658-d5b2-4892-aebf-6d831956939d.
